# Effectiveness of Natural Products—*Artemisia dubia* and Manure Digestate—On Winter Wheat Cultivation

**DOI:** 10.3390/plants14101411

**Published:** 2025-05-08

**Authors:** Ausra Baksinskaite, Modupe Olufemi Doyeni, Jurate Ramanauskienė, Dalia Feizienė, Vita Tilvikiene

**Affiliations:** Lithuanian Research Centre for Agriculture and Forestry, Instituto av. 1, Kėdainiai District, LT-58344 Akademija, Lithuania; modupe.doyeni@lammc.lt (M.O.D.); jurate.ramanauskiene@lammc.lt (J.R.); dalia.feiziene@lammc.lt (D.F.); vita.tilvikiene@lammc.lt (V.T.)

**Keywords:** digestate, biomass, soil, pesticide, weeds

## Abstract

To effectively contribute to climate change mitigation, agronomists are increasingly focused on minimizing the application of synthetic fertilizers and pesticides while ensuring that crop yield and quality are not compromised. Plant biomass and organic fertilizers are known to improve soil quality, boost plant growth, and suppress diseases. However, their overall effectiveness remains limited, hence the need for further research to enhance their agricultural performance. This study aims to explore the potential application of two natural sources (manure digestate and crop *Artemisia dubia*) for crop fertilization and protection. During the growing season, winter wheat was fertilized twice (21–25 BBCH and 30–35 BBCH) with synthetic, organic (pig manure digestate), and combined synthetic–organic fertilizers. *Artemisia dubia* biomass was incorporated before sowing and planted in strips. The soil chemical composition, crop overwintering, weediness, and diseases were assessed after two years of the respective treatments. The results showed that the organic carbon content increased by 1–5% after fertilizing winter wheat with pig manure digestate and combining fertilizers (organic and synthetic). Additionally, fertilizer or pesticide use had a significant effect on the soil pH process. Combining synthetic and organic fertilizers increased the amount of mobile phosphorus in the soil by 38%. In conclusion, combining synthetic fertilizers with organic fertilizers is the most effective approach to maintain healthy soil conditions and prevent damage to sprouts in the soil. Overall, our findings offer more opportunities for organic and sustainable agricultural processes by integrating pig manure digestate and *Artemisia dubia* biomass as a natural approach to minimizing synthetic fertilizer and pesticide use.

## 1. Introduction

As concerns over the environmental impact of synthetic inputs in agriculture continue to rise, there is growing interest in harnessing natural products to promote sustainable crop production. Notably, the biomass of non-food crops and organic fertilizers have demonstrated promising potential to improve soil health, control weeds and diseases, and enhance both crop yield and quality. At the beginning of the 20th century, Rudolf Steiner was one of the first to express concern about the adverse effects of synthetic fertilizers on soil, plant, and animal health [1]. At the end of this century, French beekeepers began to worry that certain chemicals were killing bees [2]. This has encouraged scientists to study the effects of synthetic fertilizers and pesticides on nature. The environmental movement continues to be active even now when climate change rages, and pollution from several sources affects the air, soil, and water [3]. The intensive use of synthetic fertilizers and pesticides impacts the ecosystem, promotes the loss of soil biodiversity, or even causes changes in the physical structure of the soil [4]. Although synthetic chemicals are critical to improving crop production and ensuring food security, they have immediate and long-term adverse effects on nature [5]. Due to the adverse effects of synthetic fertilizers and pesticides, there is a growing interest in sustainable agricultural practices such as organic farming, biofertilizers, and biocontrol measures to reduce synthetic materials and preserve nature [6,7].

Fertilizers are one of the most important inputs for achieving good yields and improving the quality of agricultural plants. Unbalanced fertilization leads to the deterioration of soil properties, increased water pollution, and increased greenhouse gas emissions. Synthetic fertilizers promote modern agriculture’s development, but their extensive use increases environmental pollution and negatively affects soil quality [8]. Synthetic fertilizers are quickly dissolving, so they are immediately available to plants. The use of organic fertilizers is encouraged to reduce the negative impact of synthetic fertilizers on the environment. It is a widely accepted strategy for maintaining or accumulating crop yields and soil organic carbon stocks [9]. Together with microorganisms, these fertilizers can improve soil structure, promote organic matter accumulation and plant growth, and suppress soil-borne diseases [8].

One of the most common solutions for recycling organic waste is a biological process that converts organic matter into biogas and, at the same time, produces a secondary raw material—digestate [10,11]. Digestates are a mixture of water and solids and dissolved organic and inorganic matter [12,13], and they can act as biofertilizers [13]. Digestate is rich in mineral and organic nutrients that positively affect the chemical and physical properties of the soil [12] and increase fertility [14]. The speed of action of this secondary product can be compared to synthetic fertilizers [12,13]. Mixing organic fertilizers with synthetic fertilizers is a promising alternative to standard synthetic fertilizers that will help reduce the impact of synthetic fertilizers on the environment [9]. Optimizing synthetic and organic fertilizers to increase crop yields [15] and soil organic carbon is a priority. Although using organic fertilizers is beneficial in increasing soil organic carbon and providing nutrients for crops, the increase in yield is small or marginal [9,16]. Therefore, synthetic fertilizers can supplement the supply of organic nutrients [17]. However, there is still limited information about effective agricultural management technology, appropriate organic–synthetic fertilizer ratios, and their effectiveness on crop yield and quality.

Furthermore, plant protection is crucial in increasing crop productivity by ensuring that yield and quality standards are met [18]. These are usually achieved through preventing and controlling pests, diseases, and unwanted weeds [19]. The main problem is that the systematic use of synthetic pesticides changes the biological, agrochemical, physical, and chemical properties of the soil. The effect of synthetic pesticides on soil fertility depends on the dose of the synthetic substance used and the temperature and humidity of the environment [7]. Pesticides can be divided into several types, one of which is phyto-pesticides (pesticides of plant origin) [20,21]. Phyto-pesticides contain many phytochemical compounds that cause them to act through various mechanisms. This means they break down easily and are less persistent in the environment. However, it becomes a disadvantage when the goal is to eliminate pests and weeds that appear after the application of biopesticides [22,23]. Biologically active compounds in certain plant species may be effective against specific pests, weeds, or diseases [24]. During growth and development, plants produce many secondary metabolites. In some plants, essential oils suppress the growth of neighboring plants through an allelopathic effect, giving the producing plant a competitive advantage [25]. Considering the increased density of weeds among the main crops, attempts should be made to find new allelopathic plants. Allelopathic plants produce a wide range of bioactive compounds with varying compositions and concentrations, including phenolics, terpenes, flavonoids, fatty acids, and steroids, with many exhibiting phytotoxic effects against a broad spectrum of weed species. As it inhibits their photosynthetic pathways, it can disrupt the activity of metabolic enzymes [26,27,28,29]. These compounds accumulate in the soil through plant residue decomposition, root exudation, and shoot leaching. In addition, several allelochemicals from potentially allelopathic plant species have already been isolated and shown to successfully inhibit weed germination and growth, thus being environmentally friendly. Most of these allelochemicals benefit the soil by improving nutrient concentrations and enhancing soil microbial activity [26].

One of the promising plants for allelochemicals could be *Artemisia dubia*. The *Artemisia* genus belongs to the *Asteraceae* family and is distributed in various regions of the world [30]. One such plant with a strong scent is the perennial *Artemisia dubia*. These non-food plants have antimicrobial, insecticidal properties [30,31] and phytochemical and antioxidant properties [31,32,33,34,35]. *Artemisia dubia*, like other Asteraceae family plants, is a vibrant source of biologically active compounds such as polyphenols, terpenes, and flavonoids [36]. Specifically, key compounds such as several isomers of caffeic acid, chlorogenic acid, camphor, 1,8-cineole, quercetin, and luteolin are potential bioactive compounds strongly characteristic of the highly antioxidant species in *Artemisia dubia* [32]. These bioactive compounds are essential plant secondary metabolites and have specific properties. According to Polish scientists, plants of the Asteraceae family can act as biostimulators that stimulate seed germination and protect against adverse conditions or stress [33]. Hence, this study hypothesized that two natural products, pig manure digestate and plant *Artemisia dubia* biomass, can positively influence the development of winter wheat and reduce soil degradation.

## 2. Results

### 2.1. Effect of Pig Manure Digestate and Artemisia dubia on Soil Chemical Composition

**Figure 1:**
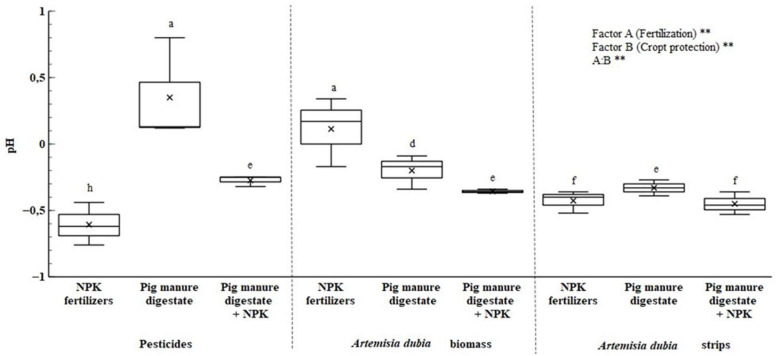
Changes soil pH after two years. Note: treatments with different letters are significantly different at *p* < 0.05. ** *p* < 0.01.

After two years of field experiments, it was observed that the soil pH mean was 14% lower than the pH values obtained before the experiment. Soil pH after two experimental years in each treatment is presented in Figure 1. Furthermore, the study results showed that using fertilizers or pesticides significantly affects the soil pH.

In the field, the soil pH value showed the highest decrease (9.7%) in the pesticides with synthetic fertilizer treatments compared to the other treatments. The lowest pH change was recorded in the treatments using pig manure digestate (170 kg ha^−1^ N) with different crop protection chosen.

Soil total nitrogen content increased in all treatments during the two years of cultivation. Statistically significant differences (*p* < 0.01) were determined in the interaction of factors A (fertilizer) and B (crop protection). The highest increase in total nitrogen content (47.2%) was recorded when *Artemisia dubia* biomass was incorporated into the soil with a combination of organic and synthetic fertilizers.

The highest levels of mobile phosphorus and potassium content were observed when *Artemisia dubia* biomass was applied with pig manure digestate. However, changes in the amount of mobile phosphorus in the soil were significant (*p* < 0.05) and depended on the fertilizer type used.

**Figure 2:**
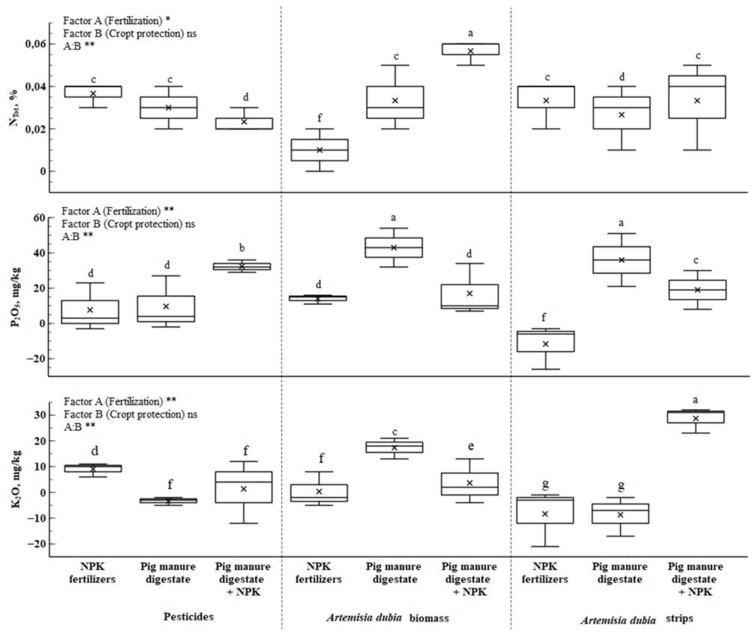
Changes in soil macronutrients (NPK) over two years. Note: treatments with different letters are significantly different at *p* < 0.05. ** *p* < 0.01, * *p* < 0.05, ns—non-significant.

Comparing the soil macronutrients showed that mobile potassium increased (Figure 2). The interaction of factors A (fertilization) and B (crop protection) showed that the most promising combination of crop protection and fertilization for increased mobile potassium content were *Artemisia dubia* biomass with digestate and *Artemisia dubia* strips with digestate and synthetic fertilizers. In these treatments, mobile potassium content increased by 12.76–24.58%. However, compared to the initial results of soil chemical elements before the experiment, total nitrogen and mobile phosphorus levels were lower.

**Figure 3:**
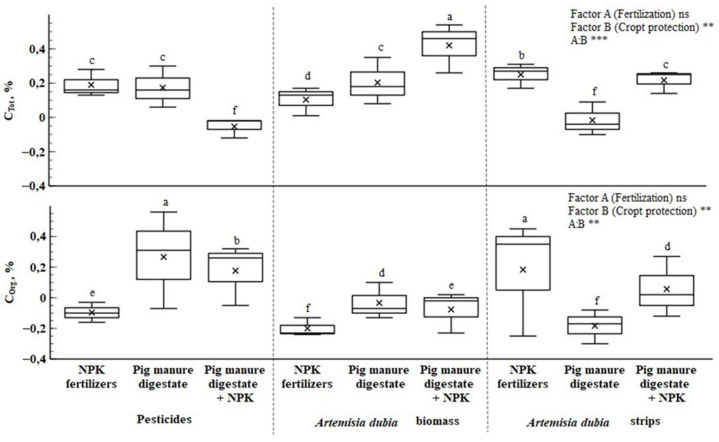
The changes of total and organic carbon in soil before and after the experiment. Note: treatments with different letters are significantly different. *** *p* < 0.001, ** *p* < 0.01, ns—non-significant.

Additionally, the total carbon content in the soil showed little change after two years of field experiments. Figure 3 shows that when winter wheat was fertilized with synthetic fertilizers, the amount of total nitrogen in the soil did not depend on the use of plant protection. The soil was significantly enriched (17.73–35.22%) in nitrogen by organic fertilizers and their combination with synthetic fertilizers. This shows that plants take nitrogen from the soil more slowly, leaving about 0.2% more total nitrogen than treatments that only use synthetic fertilizers. Although plant protection measures did not significantly affect the amount of total nitrogen, the interaction between factors A and B was statistically significant in the first field (*p* < 0.01).

The data presented in Figure 3 showed that the amount of organic carbon in the soil decreased the most when the plants were fertilized with synthetic fertilizers. The interaction of factors A and B revealed that the most promising combinations for increased organic carbon content were pesticides with pig manure digestate, pesticides with pig manure digestate and synthetic fertilizer, and *Artemisia dubia* strips with synthetic fertilizer. In these treatments, organic carbon content increased by 0.18–0.38%. This effect can be attributed to two main mechanisms. First, organic fertilizers increase soil organic carbon. Second, synthetic fertilizers contribute to this process by promoting plant vegetation, resulting in higher biomass. After harvest, the remaining plant residues decompose in the soil, increasing organic carbon levels.

### 2.2. Influence on Crop Overwintering

**Figure 4:**
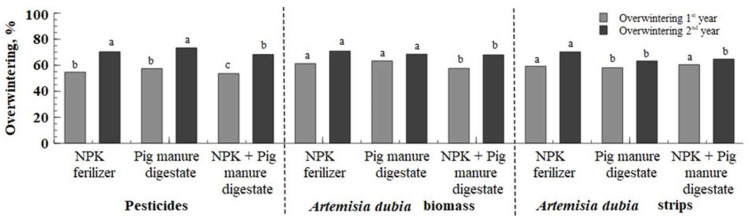
Comparisons of overwintering %. Note: treatments with different letters are significantly different.

In the first year, only the chopped biomass of *Artemisia dubia* influenced the winter wheat germination. The incorporation of chopped perennial plant biomass into the soil inhibited seed germination. This inhibitory effect could be related to the allelopathic compounds released from *Artemisia dubia* biomass, which may disrupt seed germination or hinder seedling development. However, when evaluating the crop density in spring, the results revealed that winter wheat showed the highest overwintering success when *Artemisia dubia* biomass was used (Figure 4). On average, across all fertilization treatments, the application of *Artemisia dubia* biomass led to a significantly higher crop density during spring over the experimental period. Specifically, winter wheat overwintering rates ranged from 58% to 61%.

In the second year of the study, better overwintering of winter wheat was found in pesticides and treatments with *Artemisia dubia* biomass where synthetic or organic fertilizer were used. The plants were better prepared for winter conditions in these treatments, probably due to more intensive growth and stronger winter wheat from autumn. The interaction between factors A and B showed that the most promising combinations of crop protection and fertilization for winter wheat overwintering did not result in significant differences (*p* > 0.05) in the second year of the field experiment.

Although biomass and grain quality indicators were not reported in this study, previous similar research recorded that the combined application of pesticides and pig manure digestate was the most effective treatment for maximizing both biomass and grain productivity [15].

### 2.3. Impact on Weeds and Winter Wheat Disease 

Previous studies [37] investigated the allelopathic effect of *Artemisia dubia* aboveground parts on crops and weeds. During the experiment, weed monitoring was carried out twice to analyze the effect of *Artemisia dubia* biomass incorporated into the soil, the strips planted with *Artemisia dubia*, and the herbicides used. The first weed count was carried out before the application of herbicides. During the counts, it was found that in all the studied fields, about 80% of the weeds consisted of Glechoma hederacea and Viola arvensis species. Species such as Poa annua, Galium aparine, Stellaria media, Tripleurospermum perforatum, Fallopia, and Cirsium arvense and other isolated weed species dominated.

**Figure 5:**
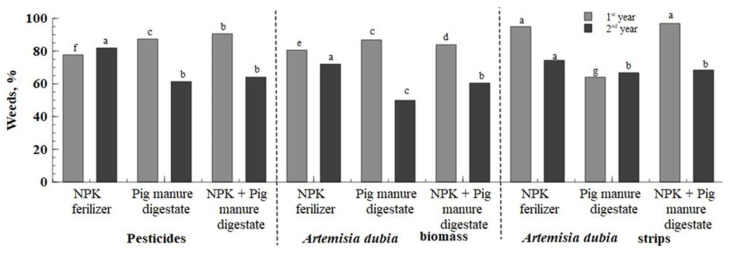
Percentage of weed control. Note: treatments with different letters are significantly different.

The results showed that in the first year of the study, the weed amount decreased significantly after herbicide application in *Artemisia dubia* strip treatments (Figure 5). The interaction of factors A and B showed that the most promising combinations of crop protection and fertilization that influenced the highest weed population decrease (86.8–96.9%) were pesticides with pig manure digestate, *Artemisia dubia* biomass with digestate and synthetic fertilizer, and *Artemisia dubia* strips with synthetic fertilizer or with digestate and synthetic fertilizer.

The germination of weeds after winter depends on meteorological conditions. The second year of study had a dry spring compared to the first year. Weed counts were conducted three weeks after herbicide application in the pesticide-only treatments. The results showed more weeds in treatments where *Artemisia dubia* biomass was applied compared to those with pesticides or *Artemisia dubia* strips. Pig manure digestate also contributed to increased weed presence, possibly due to the introduction of small weed seeds contained in the digestate.

### 2.4. Impact on Winter Wheat Disease

**Figure 6:**
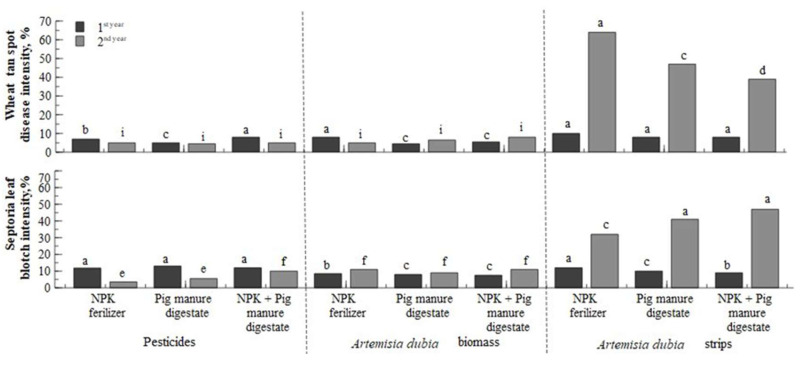
Wheat streak and septoria leaf blotch disease intensity. Note: treatments with different letters are significantly different.

During the field experiment, crop protection products had a significant effect on disease incidence. The data presented (Figure 6) showed that the incidence of wheat tan spot (*Pyrenophora tritici-repentis*) and septoria leaf blotch (*Zyniseotirua trutucu*) were influenced by the fungicides used and the incorporation of *Artemisia dubia* biomass into the soil before sowing. In these treatments, the plants were healthier in the first and second years of the study. A higher incidence of diseases was observed in the second year of the study, which showed that the pathogens associated with plant residues remained in the soil when winter wheat was transplanted. These results thus highlight crop rotation’s importance in reducing disease spread and maintaining healthy soil.

In summary, the interaction of both factors (factor A—fertilization and factor B—crop protection) did not have a statistical difference, which suggests that healthy soil may have contributed to crop resistance. The soil was enriched with nutrients and was characterized by a lower abundance of pathogens, which could have positively affected crop health and disease resistance.

## 3. Discussion

### 3.1. Soil Change After Two Years of Experiment

The soils of many European countries, including Lithuania, are characterized by a low organic matter content [34]. The use of organic fertilizers is considered vital for developing agricultural systems [35]. Digestate has an alkaline reaction, with its pH ranging from 8.0 to 8.8, so soil acidity likely decreases when fertilized with this organic fertilizer [37]. The pH value of pig manure digestate used in the study ranged from 8.00 to 8.60. Other scientists’ studies also used digestates with a similar pH value, where fixed pH values reach 7.95–9.00 [38,39,40]. However, the results show that using *Artemisia dubia* biomass with pig manure digestate or combining organic fertilizers with synthetic ones leads to soil acidification. This may be due to the biomass of *Artemisia dubia*, which is characterized by an acidic medium (pH 5.65) in the aqueous extract studies. One of the important soil parameters is its reaction. How the soil reacts to different substances determines its fertility and the formation of physical, chemical, and biological properties. In addition, the soil reaction is closely related to the solubility of minerals and the microorganisms present in it. At the beginning of the field experiment, the soil pH was neutral (6.52). The results show that pig manure digestate applied for two years can increase soil pH or at least keep it stable. Polish researchers obtained similar results and noted that their findings agreed with other researchers’ data [35]. However, in field studies, the pH value decreased or remained unchanged when digestate was combined with synthetic fertilizers. In the studies of some other scientists, when analyzing the combination of organic and synthetic fertilizers, the opposite results were obtained; according to their data, when combining fertilizers, the pH value increases, which is especially useful in acidic soils [35].

Pig manure digestate is rich in basic micronutrients, such as nitrogen, phosphorus, and other macro- and micronutrients important for plants. Using digestate can contribute to increasing the sustainability of agriculture by reducing greenhouse gas emissions associated with fertilizer production and restoring nutrient cycles [37]. In addition, it helps replenish soil nutrient reserves and increases its ability to supply plants with the necessary substances [41]. Plant-available phosphorus (P_2_O_5_) and potassium (K_2_O) are among the most important elements for growth, together with nitrogen (N), which influences not only yield but also its quality [35,37]. Like Barlog and other researchers [41], his study observed that the amount of plant-available P_2_O_5_ in the upper soil layer increased every year with the use of digestate and synthetic fertilizers. The results of the study showed that the highest amount of P_2_O_5_ available to plants was determined using digestate alone, which confirms that digestate is a rich source of phosphorus. The use of organic fertilizers can directly or indirectly influence the availability of P_2_O_5_ in the soil: directly through organic and inorganic compounds and indirectly by promoting the activity of soil microorganisms [42]. The obtained results align with the research findings of García-López [43], which confirmed the significant effect of digestate in increasing the amount of phosphorus available to plants. Additionally, digestate can serve as a valuable source of K_2_O in the soil. Studies by Barlog and other scientists revealed that the amount of K_2_O available to plants increased after using digestate in the soil [41]. However, our research has shown that using digestate leaves less mobile phosphorus in the soil than synthetic fertilizers (SF) or a combination of SF and pig manure digestate.

Nitrogen is one of the most important elements of plant nutrition, which has a decisive influence on the yield and quality [37]. The study found that using pig manure digestate alone or in combination with SF increased the total amount of N in the soil. Similar results were obtained by Spanish and Portuguese researchers [43]. It was also found that using pig manure digestate together with chopped *Artemisia dubia* biomass increased the total N content in the soil. However, part of the nitrogen supplied is not completely mineralized during one cycle [42], which is also confirmed by previous studies using nitrogen isotopes [44].

The application of different fractions of anaerobic digestion residues to soil can have unequal effects on the soil’s carbon cycle [45]. The total amount of carbon in each soil formation varies and depends on how it is used. The total amount of carbon in the soil increases by adding above-ground and below-ground plant biomass and organic residues in natural and organic fertilizers [35]. However, the effect of digestate on soil organic carbon content is not always positive, with some studies reporting no significant effect on organic carbon concentration [40], which aligned with the results of our study.

### 3.2. Effects of Applied Natural Materials on Weeds and Crop Diseases

Organic mulches effectively reduce the emergence and growth of weeds in various environments [46]. Their effectiveness depends on the mulch material type, the application’s thickness, and the specific interaction between crops and weeds [47]. Research shows that mulch primarily inhibits weed growth by limiting light availability. Although the physical properties and thickness of the mulch layer are important for weed control, some allelochemicals in the mulch can also inhibit their growth in some instances [48]. When covering the soil with mulch, the chemicals it contains can effectively reduce weed germination. However, they are not strong enough against perennial weeds [49]. In agriculture, the effectiveness of weed control depends on the ability to destroy seeds already in the soil and limit the entry of new ones into the soil. Weed seed germination is greatly influenced by temperature and humidity. Different weed species respond differently to soil temperatures, with some germinating in early spring while others only germinate after the soil has warmed considerably. Moisture is also important because it helps remove the seed coat and dissolve its nutrients. In addition, moisture is closely related to temperature, with seeds swelling most rapidly at 20–30 °C, and at lower temperatures, this process occurs more slowly [46]. As can be seen from the results obtained (Figure 6), the weeds did not completely disappear after using synthetic herbicides. This proves that weed seeds in the soil can successfully germinate when exposed to the right conditions. Meanwhile, the application of crushed *Artemisia dubia* mulch reduced weed germination due to the release of allelochemicals. *Artemisia dubia* showed the strongest allelopathic effect because it had the highest content of phenolics and flavonoids [26]. This suggests that allelochemicals can be directly extracted from *Artemisia dubia* and used as environmentally friendly bioherbicides for weed control [36,50].

Crops are constantly attacked by pathogens before and after harvest, often resulting in significant economic losses. To improve the quality and quantity of agricultural production, it is necessary to apply environmentally friendly disease management measures. Many biologically active compounds have been isolated, some of which have contributed to the development of new plant-based biopesticides [21,51]. However, the production of preparations based on biological components is complex. It requires a lot of starting material, and the final product often has a different quality due to the variable content of biologically active compounds. In addition, during the manufacturing process, some organic compounds may be lost, which may make the preparation unstable [51]. Due to constantly changing farming systems and the limited choice of alternative crops, many farmers grow the same crops in the same field for two or more consecutive years. This increases the risk of disease spreading in cereal crops [52]. Most fungal diseases appear at the initial stage in individual areas of the crop, and this is influenced by three main factors: the uneven spread of the disease through residual infections in the field, the introduction of pathogens from other areas, and the heterogeneity of the microclimate in the field. Weather conditions are one of the most important factors determining the development of disease epidemics [53]. The results of our research confirm the influence of microclimate on plant diseases. In the first year of the study, treatments using strips of *Artemisia dubia* had low disease intensity and were significantly (*p* < 0.05) different from other treatments. Strips of *Artemisia dubia* were rare and did not form a windbreak for crops. However, in the second year of the study, when the strips of *Artemisia dubia* thickened, a breeze formed, creating a favorable microclimate for the spread of pathogens. Weather conditions such as precipitation, air temperature, and solar radiation can explain up to 80% of the variation in agricultural production, but grain yields also depend on edaphic, hydrological, and agronomic factors. Rainfall that keeps the leaves moist longer is critical to successful plant infection. The longer periods of humidity facilitated the spread of pathogens, a result consistent with the findings of previous studies [51,53]. Kone and others [54] found that in humid and warm conditions, disease spores form more quickly, and therefore, the infection spreads rapidly between plants. Our research observed that *Artemisia dubia* strips, which became denser in the second year, created favorable conditions for spreading pathogens. Although there was not much precipitation during the growing seasons, the dew that formed in the mornings and the reduced air movement between the crops created an ideal environment for developing pathogens.

## 4. Materials and Methods

### 4.1. Experimental Site Description and Weather

The study was conducted in the agricultural fields of the Institute of Agriculture of Lithuanian Research Center for Agriculture and Forestry in central Lithuania, Kedainiai district. (55°23′50″ N, 23°51′40″ E), during the 2021–2023 growing seasons. In Lithuania, wheat cultivation is the dominant form of farming. Winter wheat grows from September to August. The soil is Endocalcari-Epihypogleyic Cambisol. Soil chemical composition at 0–20 cm depth before the experiment installation is shown in Table 1. The research was conducted on winter wheat (*Triticum aestivun* L.) and KWS Emilis (KWS Lochow GmbH, Bergan, Germany), and the seed rate was 210 kg ha^−1^.

**Figure 7:**
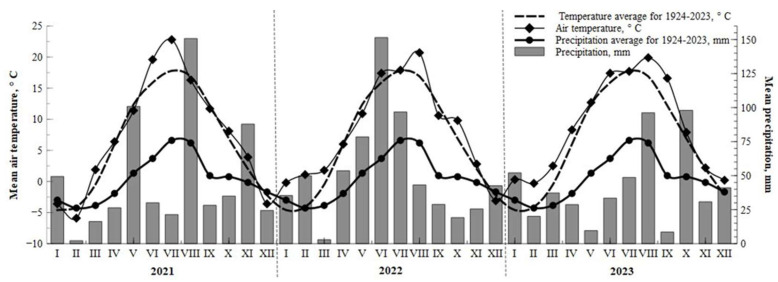
Meteorological conditions for the growing seasons 2021–2023.

The experiment started on 15 September 2021 and ran until 22 July 2023. Data of meteorological conditions from the beginning to the end of the experiment are presented in Figure 7.

Meteorological data were provided by the Lithuanian Hydrometeorological Service at the Dotnuva meteorological station.

### 4.2. Experimental Design

The field experiment was set up in the first year, 2021, on 10 September and the second year, 2022, on 19 September. It was conducted in complete randomized design order with 9 treatments of 4 replications each. Each plot was 30 m^2^ (3 m × 10 m). The study consisted of two factors—fertilization (factor A) and pesticide use (factor B) (Table 2.)

Regarding pesticide application, pesticides were found in only three treatments (experimental treatments are presented in the first paragraph of the Experimental Design section). Systemic herbicides Trimmer 50 SG (FMC Agriculture solution, Hørsholm, Denmark), Grodyl (Bayer AG, Leverkusen, Germany), and MCPA super (Nufarm GmbH & Co KG, Linz, Austria) were applied at the tillering stage of winter wheat (30–35 BBCH). When the last leaf of winter wheat appeared (37 BBCH), the fungicide Prosaro (Bayer AG, Leverkusen, Germany) was sprayed.

Regarding fertilizer application, during the vegetation period, winter wheat was fertilized twice at the beginning of vegetation (21–25 BBCH) and during the tillering stage (30–35 BBCH). Ammonium nitrate, potassium chloride, and superphosphate were used as synthetic fertilizers. Pig manure digestate was used as an organic fertilizer. Fertilization was divided into two application rates. For the first fertilization, synthetic nitrogen fertilizers and organic fertilizers were applied to the treatments according to the calculated nitrogen active substance, 90 kg N ha^−1^, and in the second stage at 80 kg N ha^−1^.

In total, 170 kg N ha^−1^ of nitrogen fertilizers were applied to the fields. Potassium and phosphorus also were applied according to the calculated existing active substances of potassium and phosphorus in pig manure digestate. The digestate was obtained from a pig farm in Lithuania. The pig manure digestate was spread on the soil surface without incorporation. Chemical analyses of pig manure digestate were performed each time before fertilization (Table 3).

Total nitrogen (N_tot_) was determined according to the Kjeldahl nitrogen distiller method, a Flame Photometer (Sherwood, Cambridge, UK) was used to determine mobile potassium, and a UV-VIS Spectrophotometer (Shimadzu, Duisburg, Germany) was used to determine mobile phosphorus.

For the *Aretemisia dubia* biomass, *Artemisia dubia* plant was grown in an energy crop plantation and was fertilized with additional nitrogen fertilizer (180 N kg ha^−1^) during the vegetation season. Three cuttings were made by chopping the *Artemisia dubia*. The second cut of the biomass was chosen for the field experiments since the highest concentration of phenols was determined during the second cut. After cutting, *Artemisia dubia* biomass was left to dry at room temperature and crushed to 3–5 cm with an electric crusher Viking GE 420 (Stihl, Vösendorf, Austria). The crushed biomass was spread into treatments of 8 t ha^−1^.

Regarding *Artemisia dubia* strips, before the sowing of winter wheat, *Artemisia dubia* plants were planted first. The strips were 1.5 × 10 m, spaced 0.5 m apart, and planted with three checkerboard trips. After winter wheat harvesting, the *Artemisia dubia* strips were cut in the autumn before the next season’s sowing.

### 4.3. Determination of Soil Chemical Composition

Soil samples were collected with a drill from the 0–20 cm layer. The soil was dried at air temperature before chemical analysis. Mobile phosphorus (P_2_O_5_) and mobile potassium (K_2_O) were determined according to Egner–Riehm–Domingo (A-L). Organic carbon (C_org_.) was determined according to [55] by dry combustion and for the determination of pH_KC_l, according to LST [56]. Total nitrogen and carbon were determined by weighing 10 mg of the soil sample into a tin capsule. The capsule was folded so that no air remained in it. The samples prepared were placed in the autosampler of the CHNS-O (ECS 4010—elemental combustion system) elemental analyzer. The samples were burned according to the principle of helium gas combustion. The results of the analyzed plant were subsequently calculated using the elemental analysis software computer program.

### 4.4. Assessment of Weediness and Diseases

The distribution and diversity of weeds were determined randomly in the field in two locations by selecting 0.25 m^2^ area plots. Weediness was identified twice: during the intensive growth of weeds in the middle of May and after the application of herbicides in June.

The accounting of the prevalence and intensity of diseases in winter wheat was carried out by the plant until the stage of wheat milk maturity. The prevalence of plant diseases was visually assessed on the top three fully twisted leaves of 10 main stems randomly selected from the field and calculated as average.

### 4.5. Statistics

The observed data were statistically processed using the R version 4.4.0 program. Visualization was provided using Veusz 3.6.2 scientific plotting software. Multiple ranges were applied to identify treatment effects and identify significant differences in alpha means at the 0.05 level.

## 5. Conclusions

The application of pig manure digestate and chopped *Artemisia dubia* biomass, both when incorporated into the soil and when planting plants in strips, had different effects on soil chemical composition indicators, weed prevalence, and disease intensity to reduce the use of synthetic fertilizers and pesticides with key findings including the following:

- Pig manure digestate affected the soil more effectively when used alone compared to the treatments in which it was combined with synthetic fertilizers or crushed *Artemisia dubia* biomass.
- The highest amount of total N in the soil was accumulated when crushed *Artemisia dubia* biomass was incorporated and combined fertilization (organic and synthetic fertilizers together) was applied.
- In treatments with incorporated *Artemisia dubia* biomass, a significant decrease in the prevalence of weeds was observed. This suggests that *Artemisia dubia* biomass can be used as a natural weed control tool as well as contribute to reducing the intensity of pathogens.

## Figures and Tables

**Table 1:** Soil chemical parameters before the experiments.

pH	P_2_O_5_ mg/kg	K_2_O mg/kg	C_organic_ %	C_total_ %	N_total_ %
6.52 ± 0.12	102.00 ± 9.12	118.00 ± 10.23	1.08 ± 0.12	1.39 ± 0.30	0.14 ± 0.01

**Table 2:** Factor A and factor B.

Factor A (Fertilization)	Factor B (Crop Protection)
Synthetic (NPK) fertilizers	Pesticides
Pig manure digestate	*Artemisia dubia* biomass
Pig manure digestate combination with synthetic (NPK) fertilizers	*Artemisia dubia* strips

**Table 3:** Digestate chemical characteristics.

	2022		2023	
	1st Fertilization	2nd Fertilization	1st Fertilization	2nd Fertilization
N_total_, %	0.240	0.315	0.216	0.272
P_2_O_5_, %	0.012	0.016	0.033	0.062
K_2_O, %	0.146	0.121	0.142	0.084
pH	8.23	8.40	8.51	8.60

## Data Availability

Data available in a publicly accessible repository.
